# The impact of coronavirus disease 2019 (COVID-19) on the antimicrobial stewardship pharmacist workforce: A multicenter survey

**DOI:** 10.1017/ash.2022.37

**Published:** 2022-04-07

**Authors:** Megan R. Wimmer, Lucas T. Schulz, Ashlee G. Hamel, Rebecca J. Schwei, Karen Fong, Donna R. Burgess, Meghan Brett, Cory M. Hale, Marisa Holubar, Rupali Jain, Rachel Larry, Emily S. Spivak, Helen Newland, Jessica Njoku, Michael Postelnick, Carla Walraven, Michael S. Pulia

**Affiliations:** 1 Department of Pharmacy, University of Wisconsin Health, Madison, Wisconsin; 2 Department of Pharmacy, Sentara Healthcare, Norfolk, Virginia; 3 Department of Emergency Medicine, University of Wisconsin Madison, Madison, Wisconsin; 4 Department of Pharmacy, University of Utah Health, Salt Lake City, Utah; 5 University of Kentucky Healthcare, Lexington, Kentucky; 6 University of New Mexico Hospital, Albuquerque, New Mexico; 7 Department of Pharmacy, Penn State Health Milton S. Hershey Medical Center, Hershey Pennsylvania; 8 Stanford University School of Medicine, Stanford, California; 9 University of Washington, Seattle, Washington; 10 Department of Pharmacy, Northwestern Memorial Hospital, Chicago, Illinois; 11 University of Utah Health, Salt Lake City, Utah; 12 Barnes-Jewish Healthcare, St Louis, Missouri; 13 Harris Health System, Houston Texas

## Abstract

**Objective:**

The coronavirus disease 2019 (COVID-19) pandemic has required healthcare systems and hospitals to rapidly modify standard practice, including antimicrobial stewardship services. Our study examines the impact of COVID-19 on the antimicrobial stewardship pharmacist.

**Design:**

A survey was distributed nationally to all healthcare improvement company members.

**Participants:**

Pharmacist participants were mostly leaders of antimicrobial stewardship programs distributed evenly across the United States and representing urban, suburban, and rural health-system practice sites.

**Results:**

Participants reported relative increases in time spent completing tasks related to medication access and preauthorization (300%; *P* = .018) and administrative meeting time (34%; *P* = .067) during the COVID-19 pandemic compared to before the pandemic. Time spent rounding, making interventions, performing pharmacokinetic services, and medication reconciliation decreased.

**Conclusion:**

A shift away from clinical activities may negatively affect the utilization of antimicrobials.

The coronavirus disease 2019 (COVID-19) pandemic placed a heavy burden on acute-care pharmacists including increased demands surrounding medication access and distribution, formulary decision making, and development and implementation of COVID-19 treatment algorithms.^1,2^ However, detailed information on exactly how the pandemic affected pharmacist work effort and activity is scarce. Furthermore, multiple reports have documented high rates of empiric antibiotic use in hospitalized patients with COVID-19 despite low rates of bacterial coinfections.^3^ This antibiotic use has raised global concerns regarding the consequences of the pandemic on the longstanding public health crisis of bacterial resistance. Although there is no doubt that antimicrobial stewardship program (ASP) pharmacists supported the pandemic response, it is unknown to what extent to which the pandemic affected non–COVID-19–related antimicrobial prescribing and how ASPs implemented stewardship interventions for patients with suspected or confirmed COVID-19. Therefore, we undertook a national survey to investigate acute-care pharmacist perspectives on the impact the COVID-19 pandemic has had on their work and antimicrobial stewardship efforts.

## Methods

### Study design and data collection

A survey was sent electronically to 474 hospitals through 4 listservs within Vizient (Dallas, TX), the largest member-driven, healthcare performance improvement company in the United States. Participants could access the survey from March 1, 2021, through April 1, 2021 (Supplement 1). Participants were not required to complete all sections of the survey, and all survey responses were included. The survey was granted a waiver of informed consent and qualified as exempt from the University of Wisconsin Madison Institutional Review Board.

Survey participants were acute-care pharmacists who self-identified as having responsibility in verifying, monitoring, and evaluating the appropriateness of antimicrobial therapy for hospitalized patients. All practicing acute-care pharmacists were included. Participants categorized their extent and type of postgraduate training, current role, and hospital setting. Participants estimated the percent of their typical workday spent completing various task, including those specific to antimicrobial stewardship, prior to and at the December 2020–January 2021 peak of the COVID-19 pandemic. Predetermined task categories were derived by consensus of the study team (Supplement 1). The survey also asked participants to subjectively indicate the impact of COVID-19 on local antimicrobial utilization and antimicrobial stewardship practices.

### Statistical analysis

Descriptive statistics were used to compare the percent responses for each survey question. We compared time spent on antimicrobial-focused tasks before and during the December 2020–January 2021 peak of the COVID-19 pandemic using paired *t* tests. All *P* values were 2-sided with *P* ≤ .05 considered statistically significant. Statistical analysis was performed using QuickCalcs GraphPad.

## Results

In total, 122 responses were received, representing at least 68 (14.3%) of 475 unique hospitals. (Table 1). All US geographical census regions were represented, with responses from 30 states, primarily in the Midwest region (42%). Most hospitals were in urban settings (58%), academic medical centers (45.5%), and had ≥400 staffed beds (55.7%). Most respondents (73.1%) described their current role as an antimicrobial stewardship or infectious diseases pharmacist. Among all respondents, 75% reported filling a leadership position in the COVID-19 response. Postgraduate training was common (74.4%), with most completing an infectious diseases PGY-2 residency (54.2%).

**Table 1.** Hospital and Survey Participant Characteristics

Characteristics	Responses, n/N (%)
**Geographic census location**	
Midwest	37/88 (42)
Northeast	10/88 (11.4)
South	32/88 (36.3)
West	9/88 (10.2)
**Setting**	
Urban	51/88 (58)
Suburban	19/88 (21.6)
Rural	18/88 (20.5)
**Bed size**	
<100 beds	9/88 (10.2)
100–399 beds	30/88 (34.1)
>400 beds	49/88 (55.7)
**Hospital type**	
Community-teaching	17/88 (19.3)
Community-nonteaching	25/88 (28.4)
Academic medical center or university	40/88 (56.8)
Federally funded	3/88 (3.4)
Critical access	2/88 (2.3)
Long-term acute care	0/88 (0)
Rehabilitation	1/88 (1.1)
Age, mean y (range), n = 52	37.9 (24–68)
**Sex**	
Male	26/56 (46.4)
Female	30/56 (53.6)
**Race**	
American Indian or Alaska Native	0/55 (0)
Asian	3/55 (5.5)
Black or African American	0/55 (0)
Native Hawaiian or other Pacific Islander	0/55 (0)
White	50/55 (91)
Other	0/55 (0)
Prefer not to answer	2/55 (3.6)
Years of practice, mean (range) (n = 86)	12.65 (0–41)
**Postgraduate training**	
Current resident or fellow	7/93 (7.5)
Completed residency or fellowship	65/87 (74.7)
Years of residency and/or fellowship mean (range), n = 96	1.26 (0–4)
Residency type^b^	
Inpatient practice (PGY-1)	43/70 (61.4)
Critical care	4/70 (5.7)
Emergency medicine	4/70 (5.7)
Infectious diseases	38/70 (54.2)
Ambulatory care	1/70 (1.4)
Solid-organ transplant	1/70 (1.4)
**Current role^a^**	
Clinical pharmacist	25/93 (26.8)
Infectious diseases/Antimicrobial stewardship	68/93 (73.1)
Pharmacist manager	15/93 (16.1)
General medicine/Hospitalist	8/93 (8.6)
Critical care	7/93 (7.5)
Emergency medicine	4/93 (4.3)
Medication safety	2/93 (2.1)
Operations	2/93 (2.1)
Cardiology	1/93 (1.0)
Solid-organ transplant	1/93 (1.0)
Central pharmacist Drug information	1/93 (1.0) 1/93 (1.0)
**Leadership role in infectious diseases/antimicrobial stewardship**	
Yes	69/92 (75)
No	23/92 (25)

Note. PGY-1, post-graduate year 1.

a Respondents could answer more than once.

b Of the 88 respondents who completed hospital demographics, 82 provided the city in which their institution was located. Of these cities, 68 were listed once. Based on this information, we determine that our sample included at least 68 unique hospitals.

Participants reported relative increases in time spent completing tasks related to medication access and preauthorization (2.1% before the COVID-19 pandemic, 6.8% during the COVID-19 pandemic; difference of 323%; *P* = .018) and administrative meeting time (34%; *P* = .067) during the COVID-19 pandemic (Table 2). Conversely, decreases in relative time spent completing patient-centered tasks, such as patient monitoring via chart review (4.3%; *P* = .056), patient-related interventions (1.7%; *P* = .428), bedside rounding (2.9%; *P* = .087), pharmacokinetics (1.1%; *P* = .238), education (1.9%; *P* = .248), and admission–discharge medication reconciliation (0.2%; *P* = .118) were reported, although these were not statistically significant. Using a 5-point Likert scale, participants reported that COVID-19 increased the overall utilization of antimicrobials (77.2%) and antimicrobials for acute respiratory conditions (80.0%) (Table 3). Participants also indicated that the inappropriate use of antimicrobials overall and for acute respiratory conditions increased (70.2% and 84.2%, respectively). Inappropriate prescribing during COVID-19 increased the volume of antimicrobial stewardship interventions (Table 3). Nearly 1 in 3 respondents identified an increase in antimicrobial prescribing errors (incorrect or inappropriate medication selection, dose route, frequency, or duration). Most respondents (73.6%) indicated increased use of broad-spectrum antibiotics, leading to more opportunities for antibiotic de-escalation. The perceived rate of adverse effects due to antimicrobials remained unchanged from the prepandemic period.

**Table 2.** Impact of COVID-19 on Antimicrobial Stewardship Programs and Pharmacist Work Distribution

Variable	Before COVID-19	Peak COVID-19	*P* Value
Infectious diseases pharmacist FTE, mean no. of respondents (range)	1.4 (0–6) n = 81	1.4 (0–6) n = 79	.958
Patients receiving antimicrobials reviewed by antimicrobial stewardship	43% (0–100) n = 82	37.2% (0–100) n = 80	.018
**Time spent on antibiotic focused tasks, %**			
Chart review patient monitoring (n = 58)	27.0	22.7	.056
Patient-related interventions (n = 59)	13.1	11.4	.428
Rounding (n = 59)	13.3	10.4	.087
Medication access/preauthorization (n = 59)	2.1	6.3	.018
Pharmacokinetics (n = 59)	10.2	9.1	.238
Admission/discharge process (n = 59)	1.6	1.4	.118
Education (n = 57)	10.0	8.1	.248
Longitudinal projects (n = 58)	13.5	13.0	.829
Meetings (n = 58)	11.4	15.3	.067
Laboratory stewardship (n = 58)	5.9	4.2	.240
Other (n = 58)	6.0	3.7	.803

Note. FTE, full-time equivalent.

**Table 3.** Perceived Impact of the Pandemic on Antibiotic Prescribing Patterns and Stewardship Activities

Variable	Greatly Decreased, %	Moderately Decreased, %	No Change, %	Moderately Increased, %	Greatly Increased, %
**Antimicrobial characteristics**					
Overall utilization of antimicrobials (n = 57)	3.5	8.8	10.5	59.7	17.5
Utilization of antimicrobials for acute respiratory conditions (n = 57)	1.8	8.8	8.8	47.4	33.5
Overall inappropriate utilization of antimicrobials (n = 57)	1.8	7.0	21.1	61.4	8.8
Inappropriate utilization of antimicrobials in acute respiratory conditions (n = 57)	1.8	8.8	5.3	61.4	22.8
Antimicrobial prescribing errors (n = 57)	1.8	0.0	68.4	26.3	3.5
Broad spectrum antibiotic use in acute care (n = 57)	0.0	3.5	22.8	54.3	19.3
Rate of hospital-acquired infections (n = 57)	0.0	3.5	47.4	38.6	10.5
Adverse effects due to antimicrobials^a^	0.0	3.5	73.7	21.1	1.8
**Infectious diseases/antimicrobial stewardship characteristics**					
Weekend or after-hour coverage (n = 57)	1.8	1.8	64.9	15.8	15.8
Volume of stewardship interventions (n = 57)	12.3	22.8	26.3	31.6	7.0
Verbal communication of stewardship interventions (n = 57)	14.0	28.1	28.1	24.6	5.3
Nonverbal or written communication of stewardship interventions (n = 57)	7.0	15.8	42.1	31.6	3.5
Rounding with clinical teams (n = 57)	8.8	33.3	40.4	10.5	7.0

a Adverse effects due to antimicrobials include but are not limited to allergic reactions, nephrotoxicity, encephalopathy, neutropenia.

Methods of stewardship interventions changed throughout the pandemic. Verbal communication of stewardship recommendations decreased in 42.1% of participants while nonverbal or written communication increased in 35.1% (Table 3). Handshake stewardship or time spent rounding with clinical teams decreased in most participants (42.1%) during the COVID-19 pandemic as many institutions limited contact as much as possible. Unique and novel intervention strategies emerged in response to the COVID-19 pandemic as 35.3% of respondents reported making interventions to providers through the electronic health record (EHR) and other electronic-based communication tools (Table 4).

**Table 4.** Novel Antimicrobial Stewardship Interventions Instituted During the COVID-19 Pandemic

Intervention	Participants, No. (%)^a^
Text message to providers	9/122 (7.4)
EHR-based communication	19/122 (15.6)
Other electronic communication	15/122 (12.3)
EHR-based clinical decision support	20/122 (16.4)
EHR-based order sets	26/122 (21.3)
Virtual stewardship consults	5/122 (4.1)
COVID-19 antimicrobial stewardship guidance team	31/122 (25.4)
Other^b^	7/122 (5.7)
No COVID-19–specific interventions	5/122 (4.1)

Note. EHR, electronic health record.

a Respondents could answer more than once.

b COVID-19 rounding teams (n = 1), provider education (n = 3), guidelines/protocols (n = 3).

## Discussion

In this survey, which sampled 122 acute-care pharmacists from across the country between March 2021 and April 2021, we found that the COVID-19 pandemic significantly affected multiple aspects of ASPs. Most notably, workload tasks shifted toward administrative tasks and away from clinical tasks. This shift away from direct patient care or clinical activities during a time of increased inappropriate antimicrobial utilization may accelerate antimicrobial resistance.

Despite all hospitals facing the same novel pathogen, our report is the first to study antimicrobial stewardship interventions during the COVID-19 pandemic. An abundance of literature describing the role of ASPs in management of drug shortages, development of local treatment protocols, optimization of antimicrobial use, and reduction in antimicrobial resistance have called these programs into action during the pandemic.^4–6^ However, little has been published on what interventions were actually adapted by ASPs. Recently, Davis et al^7^ described reconfiguration of their EHR antimicrobial stewardship module, which allowed for more efficient chart review of COVID-19 patients. Staub et al^8^ found a significant decrease in antimicrobial use after implementation of multidisciplinary clinical guidance teams. In our study, <25% of participants reported their hospital instituting EHR-based order sets, clinical-decision supports tools, or antimicrobial stewardship guidance teams specifically for patients with suspected or confirmed COVID-19. Whether the reported shift in effort toward administrative-related tasks during the pandemic detracted from engagement of ASPs in developing COVID-19 targeted stewardship interventions remains unknown. Given the overwhelming opportunities for ASPs to standardize treatment regimens in COVID-19 patients combined with constantly evolving guidance may have led to inconsistent implementation of COVID-19–specific stewardship interventions across hospitals.

As would be expected with distancing requirements and restrictions in the workplace, pharmacist communication of stewardship interventions shifted from verbal to nonverbal, with text messages and EHR-based communication. This change is concerning given the reported benefit of handshake stewardship. Face-to-face verbal interaction is useful compared to providing written suggestions in intensive care units.^9^ Furthermore, an ASP utilizing handshake stewardship in a pediatric hospital demonstrated reductions in antimicrobial use of up to 10%.^10^ The shift in ASP communication of stewardship interventions may have unfortunately contributed to the perceived overall inappropriate increased use of antimicrobials during the COVID-19 pandemic.

Consistent with concerns raised about the impact of COVID-19 on overall antibiotic prescribing in acute care, most participants reported increases in inappropriate prescribing both overall and for respiratory tract infections. An increase in broad-spectrum antibiotics and antimicrobial prescribing errors leading to an increase in stewardship interventions was also reported. At the beginning of the COVID-19 pandemic, data on the incidence of bacterial coinfection and secondary infections were lacking. The initial rationale for using antibiotics in viral infections stemmed from the experience of bacterial superinfection in influenza, in which an 11%–35% incidence of bacterial coinfection or secondary bacterial pneumonia is described in hospitalized patients.^11^ However, the incidence of bacterial coinfection in COVID-19 is much lower. Early in the pandemic, the literature describes only a 3.1% coinfection rate at diagnosis with few patients (4.7%) developing secondary bacterial infections during hospitalization.^12,13^ The inappropriate overuse of antimicrobials in COVID-19 patients is not unique to our study. A meta-analysis conducted by Langford et al^3^ reported that 70% of COVID-19 hospitalized patients received antimicrobials. Given that ASP pharmacists described spending less time on patient-centered tasks during the pandemic including reviewing stewardship patients, the use inappropriate antimicrobials may have gone unchecked. Furthermore, the subjective assessment of clinical appropriateness may have been influenced by rapidly changing guidance surrounding antimicrobial prescribing as well as lack of institutional guidelines and other clinical decision support tools reported by our participants. The negative consequences of excessive antibiotic use increase the risk of antimicrobial-related adverse effects, including *Clostridium difficile*, excess healthcare costs, and antimicrobial resistance.^13–15^ Although <25% of participants described an increase in adverse effects related to antimicrobials, the impact the pandemic on long-term antimicrobial resistance trends is still unknown.

Our study had several limitations. First, most respondents described themselves an infectious diseases/antimicrobial stewardship pharmacist, which may have led to selection bias. This group was likely more willing to complete a survey focused on antimicrobial stewardship-related tasks and characteristics. Second, COVID-19 infection rates were not at their peak across the country during the open-survey period. For example, during March and April 2021, the number of confirmed hospital admissions due to COVID-19 was only 27.8% of those experienced during the maximum in January 2021. Participants were asked to answer the survey questions based on their experience during peak COVID-19 rates at their institution. However, because the survey was distributed after January 2021, recall bias may have occurred. Since the survey assessed a percentage of time spent of various activities and was unable to quantify overall time spent on work-related activities, it is possible that some respondents spent a similar percentage of time on each activity while experiencing significantly increased total workload and overall work-related time. Notably, 31.6% of respondents reported increased workload on weekends and after-hour coverage. Third, data reported in this survey are subjective. Although it would be useful to report objective information regarding antimicrobial utilization before and at the December 2020–January 2021 peak of the COVID-19 pandemic, we sought to determine frontline pharmacist perceptions regarding these characteristics only. Further research is needed. Our survey was limited to pharmacists in the Vizient network, and response rates were lower than expected. Low response rates were anticipated, especially from Northeast and Western hospitals where they faced the greatest numbers COVID-19 cases, because this survey was distributed during the COVID-19 pandemic. Although the perspectives of all acute-care pharmacists are unknown, this is the largest survey of its kind with representation from a large number of hospitals.

In this study, we have described changes to pharmacist workload associated with the COVID-19 pandemic. Shifts away from clinical activities toward administrative tasks and variation away from proven tactics to improve antibiotic prescribing may have led to suboptimal antimicrobial utilization and increased rates of antimicrobial resistance.

## Supplementary material

For supplementary material accompanying this paper visit https://doi.org/10.1017/ash.2022.37. click here to view supplementary material

## References

[1] Kubin CJ , Loo AS , Cheng J , et al. Antimicrobial stewardship perspectives from a New York city hospital during the COVID-19 pandemic: challenges and opportunities. Am J Health Syst Pharm 2021;78:743–750. 33543233 10.1093/ajhp/zxaa419 PMC7929392

[2] Mayi BS , Mainville M , Altaf R , et al. A crucial role for antimicrobial stewardship in the midst of COVID-19. J Microbiol Biol Educ 2021;22:22.1.69. 10.1128/jmbe.v22i1.2285 PMC8060144 33953821

[3] Langford BJ , So M , Raybardhan S , et al. Bacterial coinfection and secondary infection in patients with COVID-19: a living rapid review and meta-analysis. Clin Microbiol Infect 2020;26:1622–1629. 32711058 10.1016/j.cmi.2020.07.016 PMC7832079

[4] Mazdeyasna H , Nori P , Patel P , et al. Antimicrobial stewardship at the core of COVID-19 responding efforts: implications for sustaining and building programs. Curr Infect Dis Rep 2020;22:23. 32834785 10.1007/s11908-020-00734-x PMC7332741

[5] Stevens MP , Patel PK , Nori P. Involving antimicrobial stewardship programs in COVID-19 response and efforts: all hands on deck. Infect Control Hosp Epidemiol 2020:13:1–2. 10.1017/ice.2020.69 PMC7137534 32167442

[6] Tande AJ , Stevens RW , Wermers RA , Estes LL. Levering existing strategies of medication stewardship to preserve and appropriately use critical supplies. Mayo Clin Proc 2020;95 suppl 9:S29–S32. 32948257 10.1016/j.mayocp.2020.07.001 PMC7491456

[7] Davis MW , McManus D , Koff A , et al. Repurposing antimicrobial stewardship tools in the electronic medical record for the management of COVID-19 patients. Infect Control Hosp Epidemiol 2020;41:1335–1337. 32507113 10.1017/ice.2020.281 PMC7308631

[8] Staub MB , Beaulieu RM , Graves J , Nelson GE. Changes in antimicrobial utilization during the coronavirus disease 2019 (COVID-19) pandemic after implementation of a multispecialty clinical guidance team. Infect Control Hosp Epidemiol 2021;42:810–816. 33100250 10.1017/ice.2020.1291 PMC7683821

[9] Evans B , Kosar J , Peermohamed S. Attitudes and perceptions amongst critical care physicians towards handshake antimicrobial stewardship rounds. Cureus 2019;11(12):e6419. 31988820 10.7759/cureus.6419 PMC6970093

[10] Hurst A , Child J , Pearce K , et al. Handshake stewardship: a highly effective rounding-based antimicrobial optimization service. Pediatr Infect Dis J 2016;35:1104–1110. 27254036 10.1097/INF.0000000000001245

[11] Klein EY , Monteforte B , Gupta A , Jiang W , May L , Hsieh YH. The frequency of influenza and bacterial coinfection: a systematic review and meta-analysis. Influenza Other Respir Virus 2016;10:394–403. 10.1111/irv.12398 PMC4947938 27232677

[12] Garcia-Vidal, C. , Sanjuan G , Moreno-Garcia E , et al. Incidence of coinfections and superinfections in hospitalized patients with COVID-19: a retrospective cohort study. Clin Microbiol Infect 2021;27:83–88. 32745596 10.1016/j.cmi.2020.07.041 PMC7836762

[13] Russell CD , et al. Coinfections, secondary infections, and antimicrobial use in patients hospitalised with COVID-19 during the first pandemic wave from the ISARIC WHO CCP-UK study: a multicentre, prospective cohort study. Lancet Microbe 2021;2:e354–e365. 34100002 10.1016/S2666-5247(21)00090-2 PMC8172149

[14] Spigaglia P. COVID-19 and *Clostridioides difficile* infection (CDI): possible implications for elderly patients. Anaerobe 2020;64:102233. 32593567 10.1016/j.anaerobe.2020.102233 PMC7315154

[15] Páramo-Zunzunegui J , Ortega-Fernández I , Calvo-Espino P , et al. Severe *Clostridium difficile* colitis as potential late complication associated with COVID-19. Ann R Coll Surg Engl 2020;102:e176–e179. 32803988 10.1308/rcsann.2020.0166 PMC7450442

