# Supplementary material for: The impact of coronavirus disease 2019 (COVID-19) on the antimicrobial stewardship pharmacist workforce: A multicenter survey
Source: Antimicrob Steward Healthc Epidemiol. 2022 Apr 7;2(1):e56. doi: 10.1017/ash.2022.37 (PMC9726570; doi:10.1017/ash.2022.37)
Supplement: Supplementary file 1 [file S2732494X22000377sup001.pdf]

Impact of COVID-19 on Antimicrobial Stewardship, Infectious Disease, Diagnostic Testing and Antimicrobial Use

Thank you for your interest in this survey that aims to characterize the impact COVID-19 has had on pharmacists' practice in acute care antimicrobial stewardship. In order to participate, you must be an acute care Pharmacist with responsibility for verifying, monitoring or evaluating the appropriateness of antimicrobial therapy for inpatients.

If you agree to participate, you will complete an online survey that will take approximately 20 minutes of your time. Your participation in this study is voluntary and only aggregate responses will be reported. Your responses will be confidential. You have the right to withdraw from participation at any time without penalty.

This survey is being conducted in collaboration with the Vizient network which is how we received your name. Lucas Schulz, PharmD, at University of Wisconsin Health is leading this study and you may contact him at 608-469-3576 or lschulz2@uwhealth.org If you have questions about the survey, you may contact Rebecca Schwei at 608-262-2908 or rschwei@medicine.wisc.edu.

Following completion of the survey, you will be asked to indicate if you are interested in participating in a follow up project that will assess the impact COVID-19 has had on diagnostic testing and antimicrobial utilization for acute respiratory conditions. This national Vizient research project will involve manual chart review and require approval from your institutional IRB. This is anticipated to take approximately 2 hours to complete depending on the number of patients tested for COVID-19 within the week prior to your abstraction date.

Instructions to complete the survey:

This survey will have 3 parts: 1) Questions pertaining to provider and hospital demographics, 2) Questions pertaining to your institution's Antimicrobial Stewardship program and time you spend completing various tasks related to antimicrobial stewardship in acute care and 3) Option to indicate interest in a follow up project that will occur in February 2021.

To choose a response, click on the button that corresponds to your answer. If you would like to change your answer, click on a different button.

If you start the survey and are unable to finish it, you can exit by simply closing the browser window and return to it later by clicking again on the link in your email, or re-entering the link sent to you. This will take you back to the next unanswered question.

If there is a question you leave blank, you will be informed that it was left blank. You will be required to answer most questions; however, an unknown option is generally available. Close your web browser at any time if you choose to end participation.

After the entire survey has been completed and all the data are ready to be submitted, please click on the 'SUBMIT' button on the last page. Once you click 'SUBMIT' you will not be able to re-enter the survey.

We appreciate your time and effort in assisting us by completing this web-based questionnaire.

Sincerely,

Vizient COVID-19 Antimicrobial Stewardship Project Leads
Ashlee Hamel, PharmD, Sentara Healthcare
Michael Pulia, MD MS, Director Emergency Medicine Antibiotic Stewardship, UW-Madison
Lucas Schulz, PharmD, UW Health
Megan Wimmer, PharmD, PGY2 Infectious Diseases Pharmacist Resident, UW Health

Click Next to begin the survey now.

To start we are going to ask you one question to ensure you are eligible to participate

Are you an acute care Pharmacist with responsibility for verifying, monitoring or evaluating the appropriateness of antimicrobial therapy for inpatients?

◯ Yes
◯ No

Now we will ask you a few questions about your training and your current role

Are you currently in residency or fellowship training?

◯ Yes
◯ No

How many years of residency/fellowship have you completed?

____

How many years of residency/fellowship did you complete?

____

Which type of residency/fellowship training(s) have you previously completed or are currently participating in? (Select all that apply)

- ☐ General Inpatient Practice (PGY-1 only)
- ☐ Ambulatory Care
- ☐ Drug Information
- ☐ Cardiology
- ☐ Geriatrics
- ☐ Critical Care
- ☐ Health-system Pharmacy Administration
- ☐ Infectious Disease
- ☐ Medication Safety/Use Policy Informatics
- ☐ Managed Care Pharmacy
- ☐ Nuclear Pharmacy
- ☐ Nutrition Support
- ☐ Oncology
- ☐ Pediatrics
- ☐ Solid Organ Transplant
- ☐ Psychiatry
- ☐ Emergency Medicine
- ☐ Pain and Palliative Care
- ☐ Other ______

How many years have you been practicing post training?

____

How would you describe your current role? (Select all that apply)

- ☐ Clinical Pharmacist
- ☐ Infectious Diseases/Antimicrobial Stewardship Pharmacist
- ☐ Emergency Medicine Pharmacist
- ☐ Critical Care Pharmacist
- ☐ Cardiology Pharmacist
- ☐ Solid Organ Transplant Pharmacist
- ☐ Oncology/Hematology/Heme/BMT Pharmacist
- ☐ Surgery Pharmacist
- ☐ General Medicine/Hospitalist Pharmacist
- ☐ Nutrition Pharmacist
- ☐ Pharmacist Manager
- ☐ Central Pharmacy/Dispensing/Compounding Pharmacist
- ☐ Medication Safety Pharmacist
- ☐ Operations Pharmacist
- ☐ Drug Information Pharmacist
- ☐ Psychiatry Pharmacist
- ☐ Neurology Pharmacist
- ☐ Pediatrics Pharmacist

Please list your current title(s)

____
(separate titles with semicolons)

Do you have a leadership role in your antibiotic stewardship team

- ◯ Yes, please describe ______
- ◯ No

For the following questions please respond based on the hospital where you primarily work.

In what state is your hospital located?

◯ Alabama
◯ Alaska
◯ Arizona
◯ Arkansas
◯ California
◯ Colorado
◯ Connecticut
◯ Delaware
◯ Florida
◯ Georgia
◯ Hawaii
◯ Idaho
◯ Illinois
◯ Indiana
◯ Iowa
◯ Kansas
◯ Kentucky
◯ Louisiana
◯ Maine
◯ Maryland
◯ Massachusetts
◯ Michigan
◯ Minnesota
◯ Mississippi
◯ Missouri
◯ Montana
◯ Nebraska
◯ Nevada
◯ New Hampshire
◯ New Jersey
◯ New Mexico
◯ New York
◯ North Carolina
◯ North Dakota
◯ Ohio
◯ Oklahoma
◯ Oregon
◯ Pennsylvania
◯ Rhode Island
◯ South Carolina
◯ South Dakota
◯ Tennessee
◯ Texas
◯ Utah
◯ Vermont
◯ Virginia
◯ Washington
◯ West Virginia
◯ Wisconsin
◯ Wyoming

In what city is your hospital located? ________________________________

How would you describe your setting
(Please pick the one answer that you think fits best)

◯ Urban
◯ Suburban
◯ Rural

How would you describe your hospital? (Pick one)
(Please pick the one answer that you think fits best)

◯ Community-Teaching Hospital
◯ Community-Non-Teaching Hospital
◯ Academic Medical Center or University Hospital
◯ City or County Hospital
◯ VA or US Government Hospital
◯ Critical Access
◯ Long-Term Acute Care Hospital (LTAC)
◯ Other ______

How many inpatient beds does your hospital have?
(Please pick the one answer that you think fits best)

◯ Less than 100
◯ 100-399
◯ Greater than or equal to 400

What percent of inpatient beds are staffed by a pharmacist?

____
(If you don't know put your best guess or leave blank)

How many beds are in your hospital's emergency department?

____
(If you don't know put your leave blank)

What percent of patients arriving to your emergency department are admitted?

____
(If you don't know put your best guess or leave blank)

Next, we are going to ask you a few questions about your hospital's antibiotic stewardship program

Does your hospital staff an Infectious Diseases pharmacist in-house on weeknights (after buisness-hours)?

◯ Yes
◯ No
◯ Don't Know

Does your hospital staff an Infectious Diseases pharmacist in-house on weekends?

◯ Yes
◯ No
◯ Don't Know

Is there an after-hours pager or phone number providers can use to contact an Infectious Diseases pharmacist for antibiotic decision making support or approvals?

◯ Yes
◯ No
◯ Don't Know

What hospital services do Infectious Diseases pharmacists who are involved in Antimicrobial Stewardship steward? (Select all that apply)

☐ General Medicine or Hospitalist Medicine
☐ Critical Care
☐ Surgical Critical Care
☐ Neurocritical Care
☐ Cardiology
☐ Solid Organ Transplant or Transplant Surgery
☐ Heme or BMT
☐ Oncology
☐ Neurosurgery
☐ Neurology
☐ Gynecology or obstetrics
☐ Pulmonary
☐ Orthopedics
☐ Urology
☐ General Surgery
☐ Pediatrics
☐ Other ______

Which of the following CDC core elements of hospital Antibiotic Stewardship programs does your hospital meet? (Select all that apply)

https://www.cdc.gov/antibiotic-use/core-elements/hospital.html

☐ Hospital Leadership Commitment
☐ Accountability
☐ Pharmacy Expertise
☐ Action
☐ Tracking
☐ Reporting
☐ Education

We are now going to ask you a few questions about the antimicrobial stewardship practices BEFORE the start of the COVID-19 pandemic

Before the start of the COVID-19 pandemic, how many Infectious Diseases pharmacists were staffed at your hospital? (FTE)

____
(FTE)

Before the start of the COVID-19 pandemic, what percentage of patients on antimicrobials at your institution were reviewed by an Infectious Diseases pharmacist?

____
(Percentage, best guess, if you don't know leave blank)

We are now going to ask you a few questions about current antimicrobial stewardship practices

Currently, how many Infectious Diseases pharmacists are staffed at your hospital? (FTE)	__________________________________<br>(FTE)
Currently, what percentage of patients on antimicrobials at your institution are reviewed by an Infectious Diseases pharmacist?	__________________________________<br>(Percentage, best guess, if you don't know leave blank)

For each of the following, estimate how much of your antibiotic focused work effort is spent completing the following tasks. If you do not engage in a specific activity, indicate the time spent completing this item as 0%. All categories must add up to 100 %. There are two columns so you can report for your work effort distribution before the COVID-19 pandemic (January 2020) and for the present time (mid-pandemic).

Pre COVID-19 Currently
Chart Review ______ ______
Patient-related Interventions through Prospective Audit (includes provider education) ______ ______
Patient-related Interventions through Best Practice Alerts ______ ______
Patient-related Interventions through Notes Published in Patient Charts ______ ______
Order Verification ______ ______
Admission Process (includes admission histories and/or medication reconciliation) ______ ______
Discharge Process (includes medication reconciliation and/or preparing or sending discharge prescriptions) ______ ______
Medication Access/Preauthorization (includes submitting test claims and/or preauthorizations through a patient's insurance to investigate cost. This also includes ensuring patients have access to non-formulary medications during their hospital stay and calling outpatient pharmacies to ensure a medication is stocked for patient use upon discharge) ______ ______
Antimicrobial Preauthorization (includes monitoring/auditing restricted antimicrobials and/or authorizing the use of restricted antimicrobials based on formulary recommendations) ______ ______
Pharmacokinetics (includes rationalizing dosing regimens for medications based on patient-specific factors (weight, age, clearance, etc) and or therapeutic drug monitoring (aminoglycosides, vancomycin, azole antifungals, warfain, etc). Dose adjustment based on renal function is included in this category) ______ ______
Rounding ______ ______
Learner Education (includes patient reviews, topic discussions, etc. with pharmacist students and residents) ______ ______
Patient Education ______ ______
Laboratory Stewardship (includes ordering/asking others to order appropriate labs to ensure safety and efficacy of medications. Examples include ordering serum creatinine to ensure medications are appropriately dosed if renally eliminated or creatinine kinase (CK) when patients are on daptomycin. For infectious diseases pharmacists, laboratory stewardship also includes working directly with microbiologists to improve results reporting in electronic medical record (selective reporting of susceptibility results from positive cultures) and/or providing recommendations for ordering of appropriate cultures, diagnostic labs, imaging, etc to aid appropriate antimicrobial utilization) ______ ______
Drug Policy Activities (includes developing/editing order sets, guidelines, and/or delegation protocols. Also includes review of antimicrobials for formulary review (monograph formulation) and/or information technology to create clinical decision support tools. Drug policy activities may also include antimicrobial utilization tracking and reporting) ______ ______
Literature Review ______ ______
Meetings (includes time you spend coordinating, conducting and/or participating in meetings) ______ ______
Longitudinal Projects (includes longitudinal activities outside drug policy activities and teaching. Projects may include overseeing resident and/or student projects, writing book chapters, writing/editing manuscripts, conducting national or local quality improvement projects, etc.) ______ ______
Other ______ ______

WARNING The percent effort you entered in the Pre-COVID period does not at up to 100. You entered ______%. Please re-check your entries.

WARNING The percent effort you entered in the Currently column does not at up to 100. You entered ______%. Please re-check your entries.

In your opinion, please indicate how much COVID-19 has impacted the following Antimicrobial practices at your hospital?

	Greatly Decreased	Moderately Decreased	No Change	Moderately Increased	Greatly Increased
Overall utilization of antimicrobials in acute care	○	○	○	○	○
Utilization of antimicrobials for acute respiratory conditions in acute care	○	○	○	○	○
Overall Inappropriate utilization of antimicrobials in acute care	○	○	○	○	○
Inappropriate utilization of antimicrobials for acute respiratory conditions in acute care	○	○	○	○	○
Antimicrobial prescribing errors (incorrect selection, dose, duration)	○	○	○	○	○
Broad spectrum antibiotic use in acute care	○	○	○	○	○
Rate of hospital-acquired infections	○	○	○	○	○
Adverse effects due to antimicrobials (e.g. allergic reactions, nephrotoxicity, encephalopathy, neutropenia, etc)	○	○	○	○	○

How much has COVID-19 affected the following Antimicrobial Stewardship practices in the acute care setting?

	Greatly Decreased	Moderately Decreased	No Change	Moderately Increased	Greatly Increased
Weekend or after hour stewardship coverage	○	○	○	○	○
Volume of stewardship interventions	○	○	○	○	○
Verbal communication of stewardship recommendations (i.e. handshake stewardship)	○	○	○	○	○
Non-verbal/written communication of stewardship recommendations (notes, BPAs)	○	○	○	○	○
Rounding with clinical teams	○	○	○	○	○

Please indicate if your hospital has instituted any new, COVID-19 specific antibiotic stewardship interventions (select all that apply)

☐ Text messages to providers
☐ EHR based communication tools
☐ Use of other electronic communication for stewardship recommendations
☐ EHR based clinical decision support (e.g. orders, best practice alerts)
☐ EHR based order sets
☐ Virtual stewardship consults
☐ COVID-19 antimicrobial stewardship guidance team
☐ Other ______

Please indicate how COVID-19 has impacted your

	Greatly Decreased	Moderately Decreased	No Change	Moderately Increased	Greatly Increased
Stress level	○	○	○	○	○
Workload	○	○	○	○	○
Hours worked	○	○	○	○	○
Job satisfaction	○	○	○	○	○
Time for personal growth	○	○	○	○	○
Intellectual challenge of work	○	○	○	○	○
Balance between personal and professional life	○	○	○	○	○

Overall, based on your definition of burnout, how would you rate your level of burnout?

○ I enjoy my work, I have no symptoms of burnout
○ Occasionally I am under stress and I don't always have as much energy as I once did, but I don't feel burned out
○ I am definitely burning out and have one or more symptoms of burnout, such as physical and emotional exhaustion
○ The symptoms of burnout that I am experiencing won't go away. I think about frustration at work a lot
○ I feel completely burned out and often wonder if I can go on. I am at a point where I may need some changes or may need to seek some sort of help

We are going to ask you a few questions about how you describe yourself

What is your age?

____

What is your biological sex?

- ◯ Male
- ◯ Female
- ◯ Prefer Not to Answer

Are you of Hispanic, Latino(a), or of Spanish origin?

- ◯ Yes
- ◯ No
- ◯ Don't Know
- ◯ Prefer Not to Answer

What is your race? (select all that apply)
(Check all that apply)

- ☐ American Indian or Alaska Native
- ☐ Asian
- ☐ Black or African American
- ☐ Native Hawaiian or Other Pacific Islander
- ☐ White
- ☐ Other ______
- ☐ Prefer Not to Answer

Finally, this last section asks about your interest participating in a follow up survey

By checking yes, you are indicating that you are interested in participating in the follow up study that will assess the impact COVID-19 has had on diagnostic testing, and antimicrobial utilization. This survey will require manual chart review and is anticipated to take approximately 1-2 hours to complete depending on the number of patients screened for COVID-19 pneumonia at your institution in the last 7 days. We will not link your name and contact information to any answers previously given during the survey.

◯ Yes
◯ No

If yes, please enter your first and last name ______________________________

If yes, please enter your email address ______________________________

If no, why not? ______________________________
